# Supplementary material for: Intramolecular charge-transfer enhances energy transfer efficiency in carotenoid-reconstituted light-harvesting 1 complex of purple photosynthetic bacteria
Source: Commun Chem. 2022 Oct 26;5:135. doi: 10.1038/s42004-022-00749-6 (PMC9814923; doi:10.1038/s42004-022-00749-6)
Supplement: Supplementary file 1 — Supplementary Information [file 42004_2022_749_MOESM1_ESM.pdf]

Supplementary Information

Intramolecular charge-transfer enhances energy transfer efficiency in carotenoid-reconstituted light-harvesting 1 complex of purple photosynthetic bacteria

Nao Yukihira [a,†], Chiasa Uragami [a, †], Kota Horiuchi [a], Daisuke Kosumi [b], Alastair T. Gardiner [c] Richard J. Cogdell [d], Hideki Hashimoto [a]*

[a] Department of Applied Chemistry for Environment, Graduate School of Science and Technology, Kwansei Gakuin University, 1 Gakuen-Uegahara, Sanda, Hyogo, 669-1330, Japan

[b] Institute of Industrial Nanomaterials, Kumamoto University, 2-39-1 Kurokami, Chuou-ku, Kumamoto, 860-8555, Japan

[c] Laboratory of Anoxygenic Phototrophs, Institute of Microbiology, Czech Academy of Sciences, 379 81 Třeboň, Czech Republic.

[d] Institute of Molecular, Cell and Systems Biology, College of Medical, Veterinary and Life Sciences, University of Glasgow, Glasgow G12 8QQ, Scotland, United Kingdom

† NY and CU contribute equally to this work.

* Corresponding author: Hideki Hashimoto, E-mail: hideki-hassy@kwansei.ac.jp

Supplementary Note 1

Inspection on the EET efficiency from β–apo-8'-carotenal to B880 Bchl *a* in Reβapo determined by fluorescence excitation spectroscopy.

The efficiency of EET from β-apo-8'-carotenal to B880 Bchl *a* was calculated by the ratio of the fluorescence excitation spectrum divided by the 1 – T spectrum in the carotenoid absorption region (430 – 550 nm) as shown with the solid grey-line in Fig. 3. The reason why we need to use 1 – T rather that absorbance is that absorbance is in logarithmic scale of the fraction of the exciting light absorbed by the solution. Since fluorescence intensity linearly depends on the excitation light intensity, the fluorescence excitation spectrum should be compared to the fraction of the exciting light absorbed by the solution (1-T spectrum), and not to absorbance spectrum. This issue was described in the historical works by Weber and Teale (1957) and Balke and Becker (1968). Indeed, the shape of the fluorescence excitation spectrum is nearly the same with 1 – T spectrum except for the carotenoid absorption region. This is good evidence that the EET efficiency of carotenoid to Bchl *a* is not unity in Reβapo.

In order to obtain better estimate of the EET value, we further need to consider the issue that some part of 1 – T spectrum in carotenoid area is also from BChl *a*. This consideration is illustrated in Fig. S1. In order to eliminate the effect of Bchl *a* absorption in carotenoid absorption region, the 1 – T spectrum of the carotenoidless LH1 from *Rsp. rubrum* strain G9+ (spectrum (3) in Fig. S1) was subtracted from both the 1 – T (spectrum (1) in Fig. S1) and the fluorescence excitation (spectrum (2) in Fig. S1) spectra of Reβapo. Then the EET efficiency was re-determined by the ratio of the spectrum (2) – (3) divided by the spectrum (1) – (3). The result turned out to be 77 ± 1 %, which is slightly decreased from 79 ± 1 % as has originally been determined by simply dividing spectrum (2) by spectrum (1). It should be noted here that all the previous works have been adopting the simple division of fluorescence excitation spectrum by 1 – T spectrum to calculate the EET efficiency from carotenoid to Bchl *a* in LH systems. The present analysis demonstrates the possibility that the previous studies may somewhat overestimate the EET efficiency.

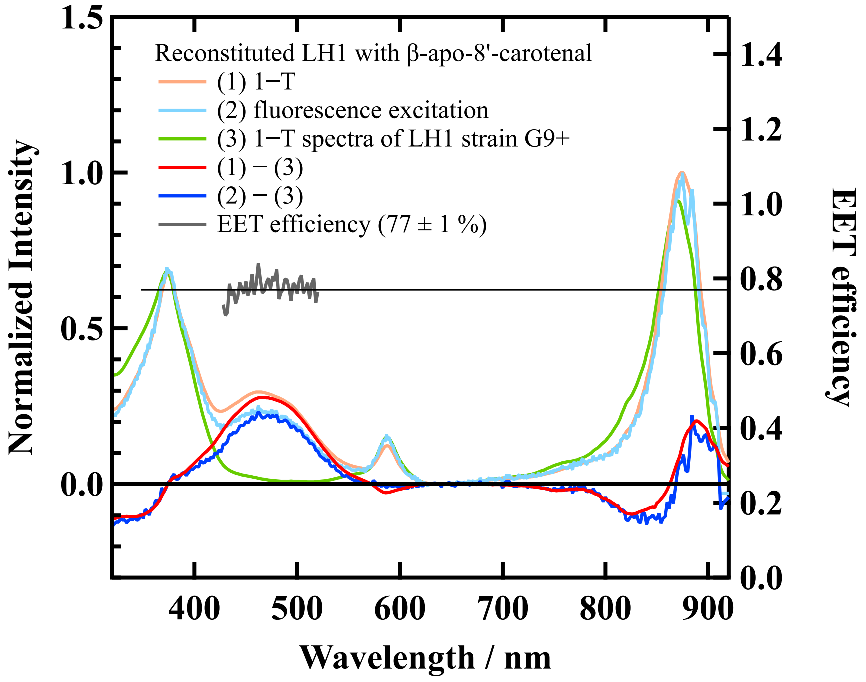

Fig. S1. Comparison of the (1) 1 – T (solid pale-pink line) and (2) fluorescence excitation (cyan line) spectra of Reβapo and (3) 1 – T (solid light-green line) spectrum of the LH1 from *Rsp. rubrum* strain G9+ (a carotenoidless strain). Spectra (1) and (2) were normalized at Q_y absorption band, while spectrum (3) was normalized at Soret absorption band. (1) – (3) spectrum was shown with solid red line, and (2) – (3) spectrum was shown with solid blue-line. The EET efficiency from β-apo-8'-carotenal to B880 Bchl *a* was calculated by the ratio of the spectrum (2) – (3) divided by spectrum (1) – (3) in the carotenoid absorption region, and the value was determined to be 77 ± 1 % (solid grey line in Fig. S1).

• G. Weber and F.W.J. Teale, Fluorescence excitation spectrum of organic compounds in solution, *Trans. Faraday Soc.* **54** (1958) 640-648.

• D.E. Balke and R.S. Becker, Relationship between the Absorption and Excitation Spectra and Relative Quantum Yields of Fluorescence of all-trans-Retinal, *J. Am. Chem. Soc.* **90** (1968) 6710-6711.

Supplementary Note 2

Discussion on the biding-site of β-apo-8'-carotenal in Reβapo.

Fig. S2 shows (a) the amino acid sequences of the LH1-α and LH1-β polypeptides and (b) the structure of LH1 subunits determined by cryo-electron microscopy (PDB ID: 7EQD) [6] from *Rsp. rubrum* strain S1. The half of a carotenoid spirilloxanthin (shown with red-color in Fig. S2(b)) is stabilized by the interaction with the hydrophobic amino-acid side-chains in the LH1-α (B870-α chain 1) and LH1-β (B870-β chain 2) heterodimer pair, and another half of spirilloxanthin is stabilized by the interaction with the hydrophobic amino-acid side-chains in neighboring LH1-α (B870-α chain 3) polypeptide and the phytol-tail of Bchl *a* (shown with blue color in Fig. S2(b)) that is coordinated to the histidine residue (His 29) of the B870-α chain 3. Polar amino acids do exist within the hydrophobic cores of both LH1-α and LH1-β polypeptides (see Fig. S2(a)). It is interesting to note that one of the methoxy groups of spirilloxanthin exits near the polar charged (basic) amino acids (Glu16 and Glu19 at the C-terminal of LH1-β). This methoxy oxygen must be stabilized by the electrostatic interaction with these polar amino acids. This idea is supported by the negatively charged feature of Glu16 and Glu 19 seen in the electrostatic potential surface shown in Fig, S2(c). If we assume that similar electrostatic interaction can also be hold true for β-apo-8'-carotenal in Reβapo, we can speculate how β-apo-8'-carotenal is bound to Reβapo. As illustrated with the chemical structures of spirilloxanthin and β-apo-8'-carotenal in Fig. S2(a), if the carbonyl oxygen interacts with and is stabilized by Glu16 and Glu19 in LH1-β, most of the polyene chain of β-apo-8'-carotenal can be stabilized by the interaction with hydrophobic amin-acids in the LH1-α and LH1-β hetero-dimer pair as has been seen in the case of spirilloxanthin. The remaining issue is the steric interaction of cyclohexene-ring of β-apo-8'-carotenal and the phytol-tail of Bchl *a*. This steric interaction may push the position of the phytol-tail away from the original biding site and may cause the steric hindrance on the binding of β-apo-8'-carotenal to the neighboring subunit. This might be the reason why only the 50% of the binding-site was occupied by β-apo-8'-carotenal when compared to the spirilloxanthin bound native LH1. The electrostatic interaction of carbonyl oxygen and polar amino-acids is reminiscent of how β-apo-8'-carotenal can be stabilized in acetone solution. β-apo-8'-carotenal is composed of hydrophobic polyene backbone and cyclohexene-ring and hydrophilic carbonyl group. When amphiphilic acetone molecules surround β-apo-8'-carotenal in solution, it is naturally expected that hydrophobic part of acetone favors the polyene and cyclohexene parts, and hydrophilic part tends to favor carbonyl oxygen. This situation is quite similar with our putative picture of how β-apo-8'-carotenal is bound to Reβapo as shown above. Therefore, we can conclude that the similar polar environmental effect must be observed for β-apo-8'-carotenal in Reβapo as has been seen in acetone based on the discussion of the structure model of LH1.

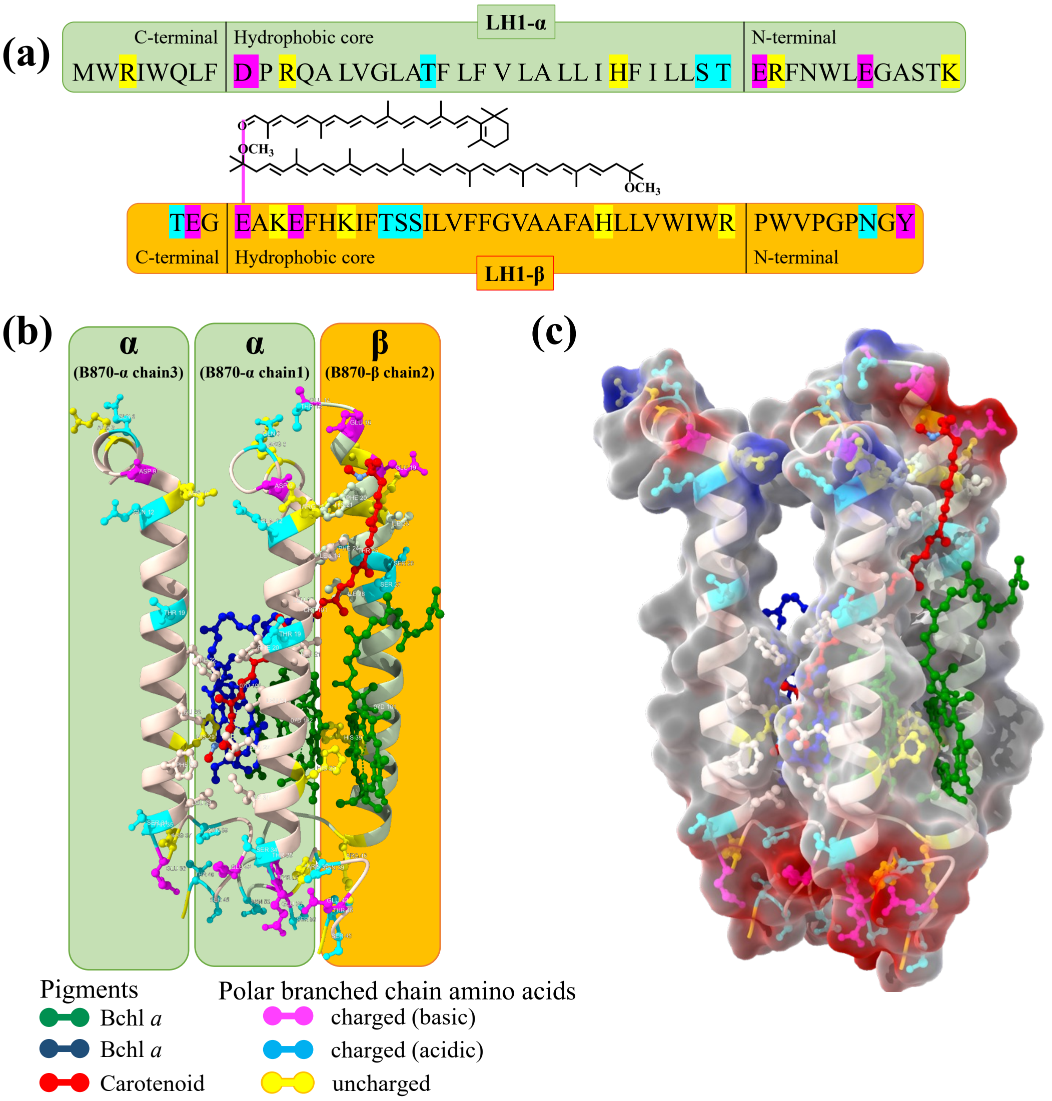

Fig. S2. (a) Amino acid sequences of LH1-α and LH1-β polypeptides from *Rsp. rubrum* strain S1. Polar charged basic, polar charged acidic, and polar uncharged amino acids were marked with magenta, cyan, and yellow, respectively. Chemical structures of carotenoids (spirilloxanthin and β-apo-8'-carotenal) are also shown. (b) Structure of the one (LH1-α and LH1-β) and a half (LH1-α) subunits of LH1 from *Rsp. rubrum* strain S1 determined by cryo-electron microscopy (PDB ID: 7EQD) [6], which was drawn with UCSF ChimelaX. Structures of the hydrophobic amin-acid side-chains that have the nearest neighbor (less than 4 Å) interaction with spirilloxanthin are shown. The side-chains of the polar charged basic (magenta), polar charged acidic (cyan), and polar uncharged (yellow) amino acids are also shown. (c) Electrostatic potential surface overlaid on the structure of the LH1 subunits. Red and blue shows, respectively, the negatively and positively charged parts. The top and bottom of (b) and (c) correspond, respectively, to the C- and N-terminals of the polypeptides.

Supplementary Note 3

Non-normalized EADS of β-apo-8'-carotenal in acetone.

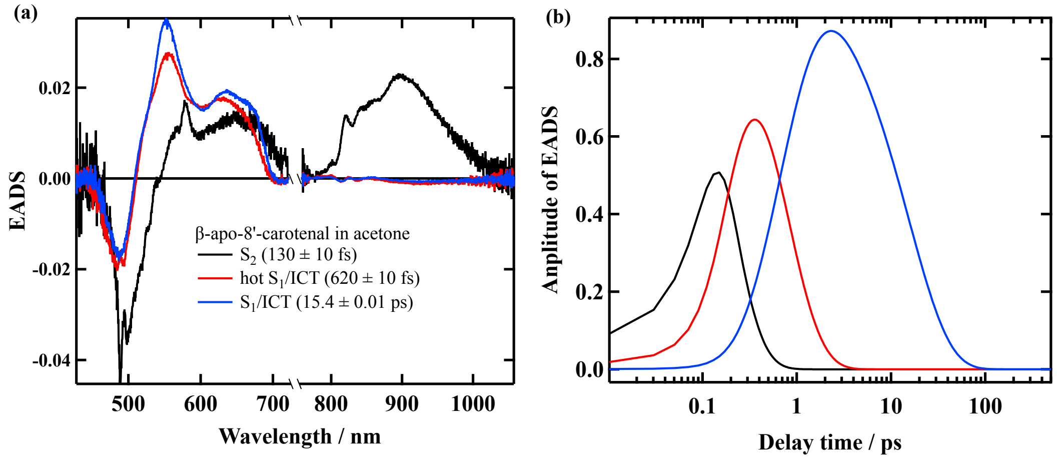

Fig. S3. (a) Non-normalized EADS and (b) their time dependence determined by the global analysis on femtosecond time-resolved absorption spectra in the visible and near infrared spectral regions of β-apo-8'-carotenal in acetone.

Supplementary Note 4

Non-normalized SADS of Reβapo.

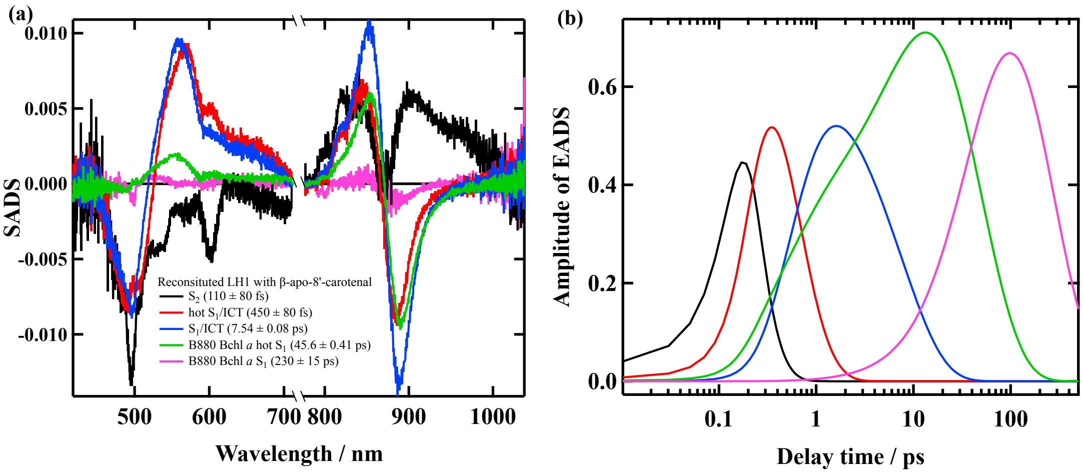

Fig. S4. (a) Non-normalized SADS and (b) their time dependence determined by the target analysis on femtosecond time-resolved absorption spectra in the visible and near infrared spectral regions of Reβapo.

Supplementary Note 5

Evaluation of the quality of the spectral fittings using a target model shown in Fig. 6(a).

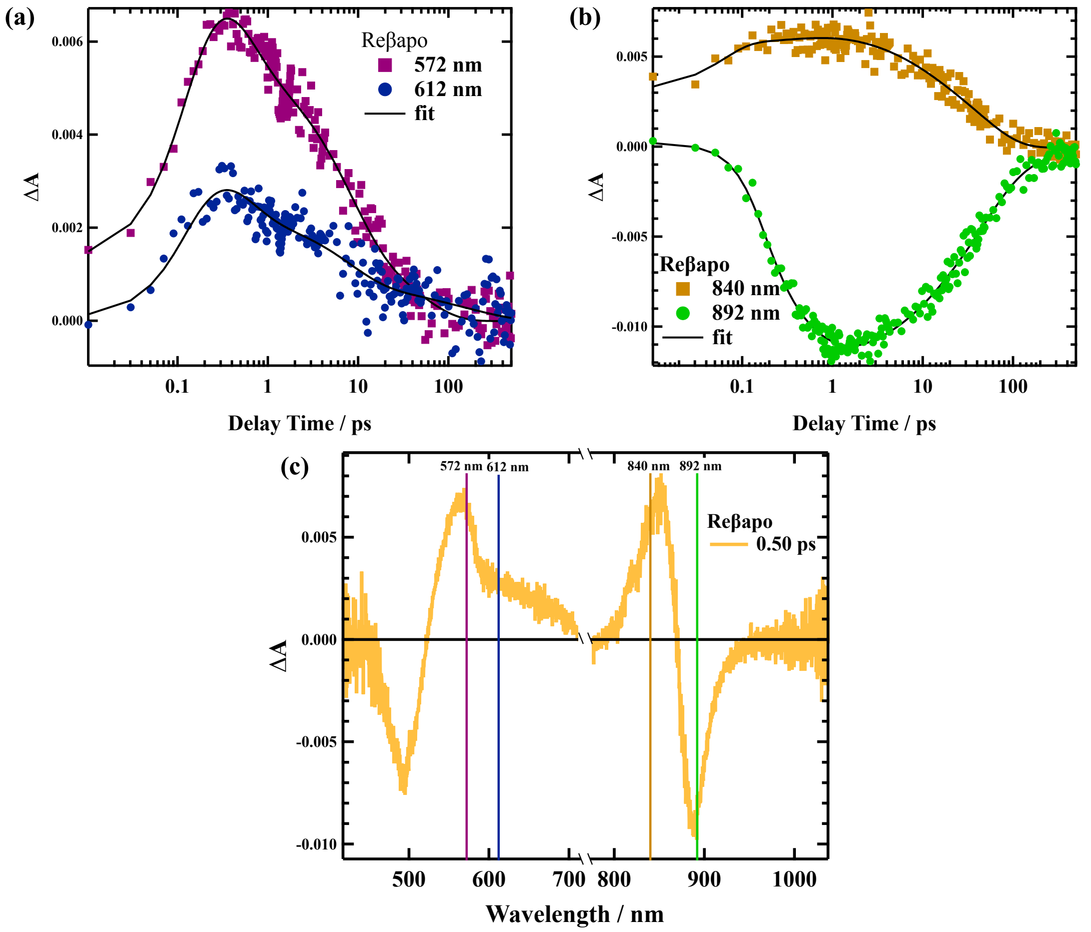

Fig. S5. Comparison of the experimentally observed kinetic traces of transient absorption of Reβapo at selected wavelengths and the results of the fittings using (a) a target model shown in Fig. 6(a) in the visible and near infrared spectral regions. The time trace at 572 nm represents the change of the 'S_1-like' excited state absorption and that at 612 nm represents the change of the ICT excited state absorption of β-apo-8'-carotenal in Reβapo, while the time trace at 840 nm represents the change of the transient absorption and that at 892 nm represents the bleaching of the Q_y band of B880 Bchl *a*. (c) Experimentally observed transient absorption spectrum of Reβapo recorded at 0.50 ps after excitation. Vertical color bars show the positions of the selected wavelengths.

Fig. S5(a) and S5(b) show that spectral fittings using a target model shown in Fig. 6(a) is quite successful.

Supplementary Note 6

Estimation of N_{eff} of β-apo-8'-carotenal.

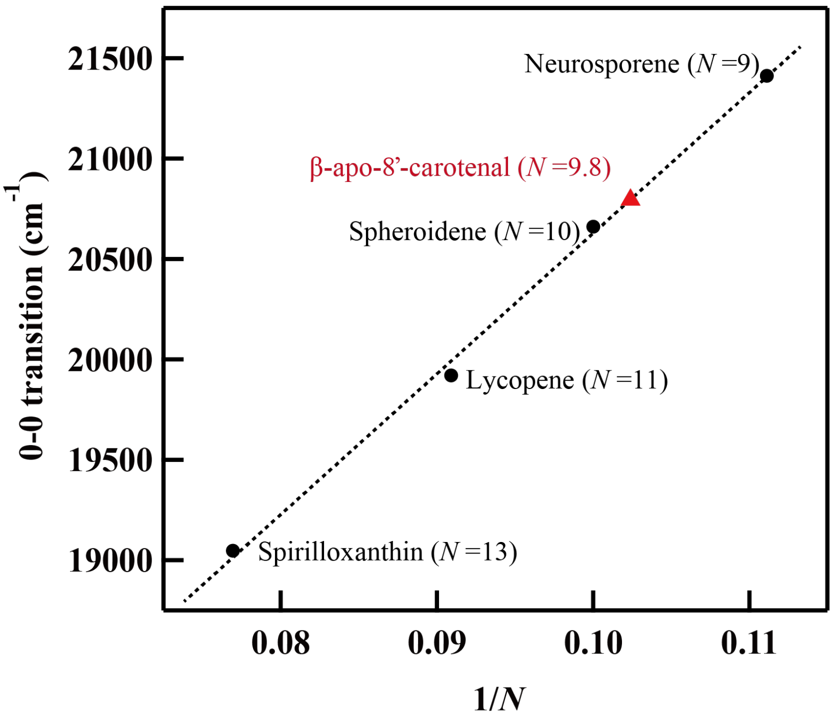

Fig. S6. Relationship between the 0-0 transition energy in cm^{-1} of the $S_0 \rightarrow S_2$ absorption and the inverse of the number of conjugated C=C bonds (1/*N*). The 0-0 transition energies of spirilloxanthin, lycopene, spheroidene, and neurosporene in *n*-hexane are reported to be 19050, 19920, 20660, and 21410 cm^{-1}, respectively [1,2].

According to this plot, the effective conjugated chain length N_{eff} of β-apo-8'-carotenal can be determined to be 9.8, which is slightly less than spheroidene (N = 10) but greater than neurosporene (N = 9).

1. Mendes-Pinto, M. M. et al. Electronic Absorption and Ground State Structure of Carotenoid Molecules. *J. Phys. Chem. B* **117**, 11015-11021, DOI: 10.1021/jp309908r (2013).
2. Araki G., Murai T. Molecular Structure and Absorption Spectra of Carotenoids, *Prog. Theor. Phys.* **8**, 639－654, DOI: 10.1143/ptp.8.639 (1952).

Supplementary Note 7

Support for the presence of EET from the hot S_1/ICT and S_1/ICT states of β-apo-8'-carotenal to the Q_y state of B880 Bchl *a*.

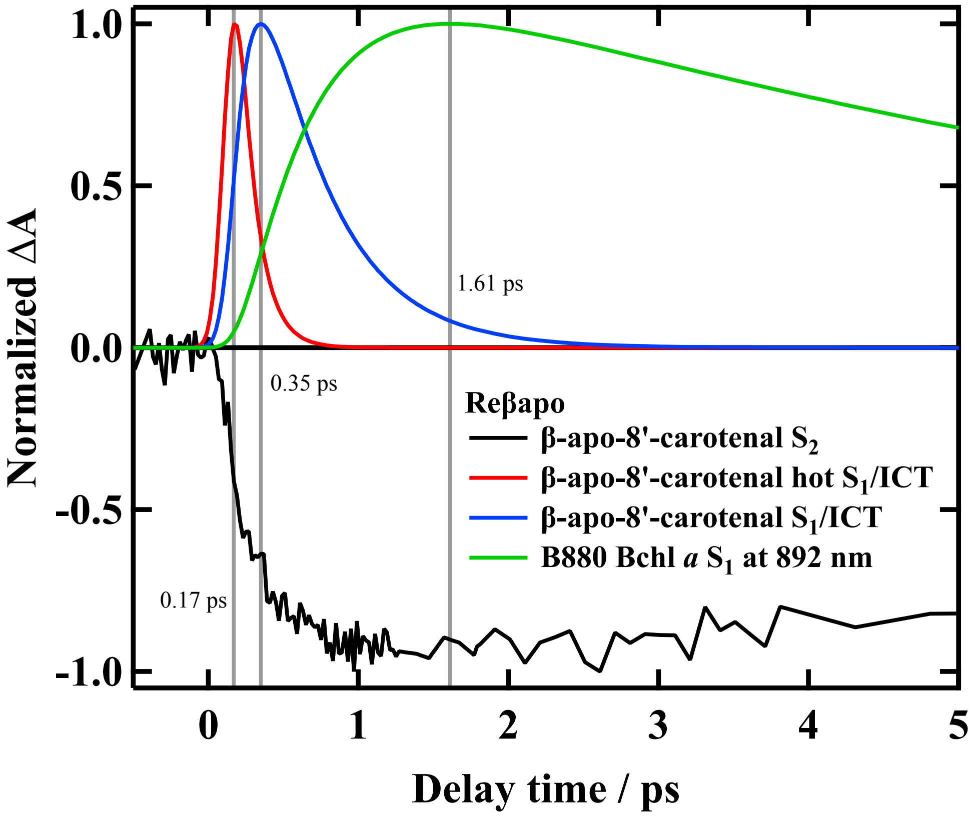

Fig. S7. Upper panel shows the normalized temporal profiles of the population of the S_2 (110 ± 80 fs), hot S_1/ICT (450 ± 80 fs) and S_1/ICT (7.54 ± 0.08 ps) components in Fig. 6(b) of β-apo-8'-carotenal in Reβapo determined by the target analysis shown in Fig. 6(a), where the convolution is carried out by taking the instrumental response function into consideration. Lower panel shows the time trace of the transient absorption spectrum of Reβapo experimentally observed at 892 nm, where bleaching of the Q_y band of B880 Bchl *a* is observed.

The rise of the time trace at 892 nm, which corresponds to the bleaching of the Q_y band of the B880 Bchl *a*, is relatively slow and is seen to be bi-phasic. The fast-rise bleaching component that is observed before 0.35 ps after excitation shows good agreement with the decay of the S_2 component of β-apo-8'-carotenal in Reβapo, while the slow-rise bleaching component observed after 0.35 ps after excitation and last until 1.61 ps after excitation shows good agreement with the decay of the hot S_1/ICT and S_1/ICT components of β-apo-8'-carotenal in Reβapo. This is a good indication that the EET from the S_2 state to the Q_x state of B880 Bchl *a* and the hot S_1/ICT and S1/ICT states to the Q_y state of B880 Bchl *a* is taking place in Reβapo.

Supplementary Note 8

The details of the calculation of what percent of the S_1 B880 Bchl *a* transforms into the T_1 state and the rates of intersystem crossing from the S_1 B880 *a* to T_1 B880 *a* (k_{isc}) and the nonradiative and radiative relaxation (k_R) from the S_1 B880 Bchl *a* to the ground state.

According to the Global analysis against sub-nanosecond time-resolved absorption spectra, we could determine the rate of intersystem crossing from the S_1 B880 Bchl *a* to T_1 B880 Bchl *a* to be

$$k_1 = k_{isc} + k_R = (230 \pm 15 \text{ ps})^{-1}.$$

Here, k_{isc} is the true rate of intersystem crossing from the S_1 B880 Bchl *a* to T_1B880 Bchl *a*, and k_R is the rate of nonradiative and radiative relaxations of the S_1 B880 Bchl *a*. Based on the amplitudes of EADS shown in Fig. 9, the rate of excitation that is delivered to the T_1 B880 Bchl *a* from S_1 B880 Bchl *a* is calculated to be

$$\frac{1.00}{1.00 + 12.0} \times 100 = 7.7\ (\%).$$

Therefore, the rate of k_{isc} and k_R can be determined as shown below.

$$k_{isc} = (230 \pm 15 \text{ ps})^{-1} \times 0.077 = (2.99 \pm 0.19 \text{ ns})^{-1}$$
$$k_R = (230 \pm 15 \text{ ps})^{-1} \times 0.923 = (249 \pm 16 \text{ ps})^{-1}$$
